# Truncated-semaphorin3A is a potential regulatory molecule to restore immune homeostasis in immune-mediated diseases 

**DOI:** 10.3389/fphar.2022.1085892

**Published:** 2023-01-10

**Authors:** Nasren Eiza, Ofra Kessler, Adi Sabag, Gera Neufeld, E. Yvonne Jones, Zahava Vadasz

**Affiliations:** ^1^ The Proteomic Unit, Bnai Zion Medical Center, Haifa, Israel; ^2^ Cancer research center, The Rappaport Faculty of Medicine, Technion, Israel Institute of Technology, Haifa, Israel; ^3^ The Division of Structural Biology (STRUBI), Nuffield Department of Clinical Medicine, Oxford, United Kingdom

**Keywords:** semaphorin3A, CD72, immune-regulation, autoimmunity, lymphocytes, cytokines, new-agent, therapy

## Abstract

Regulatory molecules have recently been recognized for their beneficial effects in the treatment of immune-mediated diseases, rather than using cytotoxic immune-suppressing drugs, which are associated with many unwanted side effects. Semaphorin3A (sema3A), a unique regulatory master of the immune system, was shown to be decreased in the serum of systemic lupus erythematosus (SLE) patients, in association with disease severity. Later, we were able to show its extremely beneficial effect in treating lupus nephritis in the NZB/W mice model. The mechanisms by which sema3A maintains its regulatory effect is by binding the regulatory receptor CD72 on B cells, thereby reducing the threshold of BCR signaling on B cells and reducing the production of pro-inflammatory cytokines. The aim of this study was to generate a stable sema3A molecule, easy to produce with a higher binding capacity to CD72 receptor rather than to Neuropilin-1 (NRP-1) receptor, which is expressed in many cell types. Using the crystallographic structure of parental sema3A, we synthesized a new secreted (shorter) sema3A derivative, which we called truncated sema3A (T-sema3A). The new molecule lacked the NRP-1 binding domain (the C-terminal site) and has an artificial dimerization site at position 257 (serine residue was exchanged with a cysteine residue). To facilitate the purification of this molecule we added Histidine epitope tag in frame upstream to a stop codon. This construct was transfected using a viral vector to 293HEK cells to generate cells stably expressing T-sema3A. T-sema3A is shown to be with a higher binding ability to CD72 than to NRP-1 as demonstrated by a homemade ELISA. In addition, T-sema3A was shown to be a regulatory agent which can induce the expression of IL-10 and TGF-β and reduce the secretion of pro-inflammatory cytokines such as IL-6, IFN-γ, and IL-17A from human T and B-lymphocytes. Keeping this in mind, T-sema3A is highly effective in maintaining immune homeostasis, therefore, becoming a potential agent in restoring the regulatory status of the immune system in immune-mediated diseases.

## Introduction

Semaphorins are a large family of transmembrane and secreted phylogenetically conserved proteins. Their receptors belong to both the neuropilins and plexins families (Tamagnone and Comoglio, 2000), through which they induce axon growth, cytoskeleton reorganization, and cell migration (Moreau-Fauvarque et al., 2003; Zhang et al., 2013). Subsequently, it became clear that they have an additional role in the process of angiogenesis, vascularization, and cancer progression. However, recent studies have indicated that semaphorins are strongly involved in the regulation of immune responses, and the restoration of immune tolerance, specifically of immune-mediated diseases such as rheumatoid arthritis (RA) and systemic lupus erythematosus (SLE) (Takegahara et al., 2005). One of the widely studied immune semaphorins in this respect is semaphorin3A (sema3A). Sema3A is well reported as a potent immuno-regulator during the early initiation and the late phase of inflammatory processes, by inhibiting T cell proliferation, migration, and pro-inflammatory cytokine secretion (Vadasz et al., 2010). The administration of sema3A to animal models of RA reduced pro-inflammatory cytokine release (IFN-γ and IL-17) and increased IL-10 secretion from CD4^+^NRP-1^+^T cells (Catalano, 2010). Sema3A serum levels from SLE patients were found to be significantly lower than in normal individuals and inversely correlated with disease activity, mainly with renal damage (Vadasz et al., 2012). In addition, low levels of sema3A in renal biopsies taken from patients with lupus glomerulonephritis were inversely correlated with proteinuria and kidney function tests (Vadasz et al., 2011). In a subsequent study conducted by our group, we were able to show that sema3A could reconstruct the B cell regulatory function by up regulating the CD72 expression on both B cells from SLE patients and healthy individuals (Vadasz et al., 2014). This study pointed to the role of sema3A in maintaining regulatory mechanisms by binding to CD72.

CD72 (a 45 kDa type II transmembrane glycoprotein) is a B cell co-receptor, expressed as a homodimer on all B cell maturation stages except plasma cells and functions as an inhibitory co-receptor, and negatively regulates B cell receptor (BCR) signaling (Nitschke and Tsubata, 2004). CD72 has a significant role in developing SLE-like disease in animal models; CD72 deficient mice (CD72^−/−^) have enhanced BCR-mediated signals that lead to more proliferative and autoreactive B cells, highly producing autoantibodies (anti-nuclear and anti–ds DNA) and spontaneously develop lupus-like disease features when they age (Li et al., 2008).

We have recently demonstrated that sema3A acts as a functional ligand for CD72 in B cells. In this study, we demonstrated that by binding CD72 the phosphorylation of STAT-4 and HDAC-1 was down-regulated, whereas P38MAPK and PKC-theta phosphorylation was up-regulated. This indicated that sema3A is a potential regulatory molecule able to restore immune-tolerance in SLE patients (Eiza et al., 2022). Considering these regulatory properties, sema3A may become a potential therapeutic agent in restoring self-tolerance.

A possible unwanted effect following a future administration of sema3A, and its binding to Neuropilin-1 (NRP-1) receptor on endothelial and neuronal cells, is the potential initiation of many complex responses such as changes in cytoskeletal organization and apoptosis of target cells (Shirvan et al., 1999; Neufeld and Kessler, 2008). In light of this, we planned to create a new synthetic sema3A, highly specific in binding CD72, to be more stable and easier to generate. In this study we aim to describe this molecule called truncated-Sema3A (T-sema3A), demonstrating its stability and functional effects in reducing pro-inflammatory properties.

## Materials and methods

### Cell lines

- HEK293-FT, human embryonic kidney cells (R70007, Invitrogen), were used for lentiviruses production.
- HEK293, human embryonic kidney cells (CRL-1573™, ATCC), were used for recombinant protein expression.
- HUVECs (human umbilical vein derived endothelial cells), primary endothelial cells isolated from the umbilical cord. These cells were isolated from a fresh umbilical cord as previously described (Jaffe et al., 1973). (These cells were not used beyond passage 8).

A *mycoplasma* detection test was routinely performed in all cell lines using the EZ-PCR™ *Mycoplasma* detection kit according to the manufacturer’s instructions (20-700-20, Biological Industries).

### Cell culture media

- HUVEC cells were grown in M-199, Hank’s salt medium (01-085, Biological Industries), supplemented with 20% Fetal Bovine Serum (10270106, Gibco™), 1% L-Glutamine 200 mM (03-020, Biological Industries), 1% MEM Vitamin Solution (100X) (01-326, Biological Industries), 0.1% Amphotericin B Solution (03-028, Biological Industries), 0.1% Gentamycin Sulfate Solution (03-035, Biological Industries), 5 μg/mL Heparin Sodium Salt (H7005, Sigma) and 5 ng/mL basic FGF (produced and purified as previously described, (Neufeld and Gospodarowicz, 1988; Tessler and Neufeld, 1990)).
- HEK293 and HEK293-FT cells were grown in Dulbecco′s Modified Eagle′s Medium (DMEM), (D5796, Sigma-Aldrich), supplemented with 10% Fetal Bovine Serum, 1% L-Glutamine 200mM, 0.1% Amphotericin B Solution and 0.1% Gentamycin Sulfate Solution.
- Primary isolated human CD22+B cells and CD3+T cells were cultured in RPMI-1640 Medium, (D5796, Sigma-Aldrich), supplemented with 10% Fetal Bovine Serum, 1% L-Glutamine 200mM, 0.1% Amphotericin B Solution and 0.1% Gentamycin Sulfate Solution.

### Cloning of truncated-sema3A (T-sema3A) construct

Following the NEBuilder HiFi DNA assembly technique (New England BioLabs, Inc.), the sequence encoding the amino acids 1-516 of the human parental sema3A (P-sema3A) was cloned into NSPI viral vector, (kindly given by Dr. Gal Akiri, (Shapiro et al., 2013)). For this cloning, three sets of PCR primers were designed as followed: the first set overlapped the 5′ region of the gene and the 3′ region of the cloning vector. The second set amplified the middle of the gene, so a point mutation S257C was introduced to allow dimerization of the protein by disulfide (S-S) bond, and the third set of primers overlapped the 3′ region of the gene and the 5′ region of the target vector, and in the 3′region of the truncated sema3A, 8xHistidine (His) tag was introduced. The three products of PCR reactions were assembled with a NSPI viral vector at 50°C for 1 h, according to the manufacturer’s instructions (NEBuilder HiFi DNA Assembly Master Mix, E2621, New England BioLabs, Inc.).

The assembly product was transformed into E. coli DH5-alpha heat shock competent cells (K12 strain) (959758026600, Bio-Lab), according to the manufacturer’s instructions. Briefly, the mixture was incubated on ice for 30 min, followed by heat shock in a 42°C water bath for 1 min, and cooling on ice for extra 5 min. Then, the mixture was spread on LB-Ampicillin-Agar plates for overnight incubation at 37°C. Screening for plasmids with positive insert was performed by Sanger sequencing at the Biomedical Core Facility, Technion, Israel, and the analysis of the sequencing and alignment with the desired sequence were performed using SnapGen^®^ 5.1.5 software.

Production of lentiviruses with T-sema3A construct and generating HEK293 cells stably expressing T-sema3A were performed as described previously (Varshavsky et al., 2008).

### Purification of sema3A

HEK293 cells produced either recombinant P- or T-sema3A fused with a Histidine tag (His) were grown at 80% confluence and cultured for 48 h in a serum-free medium. The medium was collected, filtrated, and loaded on Ni-NTA agarose beads column (30210, QIAGEN), followed by 2 times wash with a wash buffer (50 mM phosphate buffer pH-8 + 100 mM NaCl) and then eluted five times with elution buffer (wash buffer+150 mM imidazole (I0250, SIGMA)).

To assess the concentration of the purified proteins, they were subjected to SDS-PAGE along with a series of concentrations of BSA (Bovine Serum Albumin Fraction V, 160069, MP). Afterward, the SDS-PAGE was stained with InstantBlue^®^ Coomassie stain (ab119211, Abcam), and the protein amounts were assessed based on the BSA standard curve.

To verify the size of the purified proteins, they were subjected to SDS-PAGE with or without the redox agent, Dithiothreitol (D5545, Sigma-Aldrich) and immunoblotted with 0.1 μg/mL polyclonal goat anti-human semaphorin3A antibody (directed against Lys26-Val771), (AF1250, R&D Systems), followed by 1:3000 donkey anti-goat IgG (H + L)-HRP antibody (705036147, Jackson Immuno Research Labs). The bound antibodies were visualized using the EZ-ECL method (20-500, Biological Industries), and the blots were subsequently viewed by ImageQuant LAS 4000 machine and analyzed using ImageQuant TL Analysis software.

### Cell contraction assay

HUVEC cells were seeded on gelatin-coated 12-well dishes at a concentration of 4 × 10^4^ cells/well overnight at 37 °C and 5% CO_2_ in a humidified incubator. On the day of the experiment, the cells were incubated with 200 ng/mL of purified P- or T-sema3A or an appropriate amount of a control elution buffer for 30 min at 37°C. The cells were photographed using a phase-contrast inverted microscope and manually counted.

### Quantitative real-time cell contraction assay

HUVEC cells were seeded on fibronectin-coated E-plates and placed in an xCELLigence real-time cell analyzer (RTCA) overnight at 37 °C and 5% CO_2_ in a humidified incubator. On the day of the experiment, 200 ng/mL of purified P- or T-sema3A or an appropriate amount of a control elution buffer was added to the wells (time 0), and the electrical impedance through the E-plates electrodes, (which was displayed and recorded as Cell Index (CI)) was measured in real-time for 30 min as an indication of cell morphology changes.

### Sema3A and CD72 homemade ELISA

For this assay, we used a CD72 construct, which was generated by the amplification of the extracellular part of CD72 (amino acids 117-359) in a NSPI lentiviral vector with 8xHis tag. Generating HEK293 stably expressing CD72 by lentivirus infection was performed as described previously (Varshavsky et al., 2008), and the purification of CD72 recombinant protein was done using Ni-NTA agarose beads column, as descried above.

5 μg/mL purified CD72 was coated in an F96 MaxiSorp plate (442404, Nunc-Immuno) overnight at 4 °C. The next day, the plate was washed twice with PBS+0.05% Tween-20, followed by blocking with a PBS+1%BSA solution for 1 h, and then 0-25 μg/mL of purified P-sema3A or T-sema3A was added for 3 h at 4 °C. Following an intense wash process, 1 μg/mL of polyclonal goat anti human-semaphorin3A antibody (directed against Lys26-Val771) (AF1250, R&D Systems), was added for 2 h at room temperature followed by (1:10,000) anti-goat horseradish peroxidase antibody (A5420, Sigma-Aldrich) for 1 h, then, TMB-Plus substrate chromogen (S1599, Dako) for 30 min, and a stop solution (10% H_2_SO_4_). The following control conditions were evaluated as well: CD72 background (5 μg/mL CD72 without P- or T-sema3A), anti-sema3A antibody background (no CD72 nor P- nor T-sema3A were added), anti-goat HRP antibody background (5 μg/mL CD72 with 5 μg/mL P- or T-sema3A, without anti-sema3A antibody), and blank (solutions only).

The optical density (O.D.) was measured at 450nm and 360 nm using an ELISA reader and Gen5 software. The O.D. of each sample was calculated without O.D. background (the sum of all the above-mentioned control conditions). Next, the binding curves were drawn using GraphPad Prism software, and the Kd (equilibrium dissociation constant) and Bmax (maximal binding) were analyzed according to one site total binding equation.

### Stability assay

One µg of purified P- or T-sema3A was incubated in 100 µL elution buffer in a 96-well plate at a 37°C incubator for 0-72 h. At the indicated time point, proteins were collected on ice, and a sample buffer (without Dithiothreitol redox reagents) was added. All the samples were then loaded on SDS-PAGE gel, followed by electrophoresis and a transfer process. The membrane was incubated overnight with 0.1 μg/mL polyclonal goat anti-human semaphorin3A antibody, directed against Lys26-Val771 (AF1250, R&D Systems), followed by 1:3,000 donkey anti-goat IgG (H + L)-HRP antibody (705036147, Jackson Immuno Research Labs). Bounded antibodies were visualized using the EZ- ECL detection reagents (20-500, Biological Industries), with the blot being viewed by an ImageQuant LAS 4000 machine and analyzed using ImageQuant TL Analysis software.

### Isolation of primary lymphocytes

Forty mL of peripheral blood samples from six individual volunteers were drawn to heparin-washed tubes and then loaded on Lymphoprep - a Ficoll gradient (07851, STEMCELL Technologies Inc.) and centrifuged at 800xg for 30 min. The peripheral blood mononuclear cells (PBMCs) were collected and washed twice with PBS. Primary CD3+T cells or CD22^+^ B cells were isolated from PBMCs using MACS microbeads and an MS column (CD3 microbeads 130-050-101, and CD22 microbeads 130-046-401), according to the manufacturer’s instructions.

The purified CD3^+^ T cells were cultured in a 12-well plate (1x10^6^ cells/1 mL medium/well), pre-coated with 10 μg/mL anti-CD3 (CD3 Monoclonal Antibody (UCHT1), 16-0038-85, eBioscience™) for 4 h at 37°C, in addition to 1 μg/mL anti-CD28 (CD28 Monoclonal Antibody (CD28.2), 16-0289-85, eBioscience™), for 24 h at 37°C. The positively isolated B cells were cultured in a12-well plate (1x10^6^ cells/1 mL medium/well) and activated with 1 µM TLR9 agonist-CpG-ODN (ODN 2006 Class B CpG oligonucleotide, tlrl-2006-1, InvivoGen) and 5 μg/mL CD40L (Recombinant Human CD40 Ligand, 6245-CL, R&D Systems) at 37 °C for 24 h. After 24 h of incubation, 1 μg/mL of P- or T-sema3A or an appropriate amount of a control elution buffer was added for an additional 24 h at 37°C.

### Cytokines expression evaluation by flow cytometry

Activated CD22+B and CD3+T cells, stimulated with either P- or T-sema3A were harvested and fixed with Fix and Perm medium A for 10min. Afterward, cells were washed and permeabilized using Fix and Perm medium B (GAS004, Invitrogen) supplied with fluorescence antibodies for 30 min. The levels of the cytokines were evaluated in a NAVIOUS EX flow cytometer (Beckman Coulter), and the results were analyzed using Kaluza Analysis Software 2.1 (Beckman Coulter).

### Flow cytometry antibodies

• APC Rat Anti-Human IL-10, 554707, BD Pharmingen™.
• APC Rat IgG2a, κ Isotype Control, 553932, BD Pharmingen™.
• PE Mouse Anti-Human IFN-γ, 559327, BD Pharmingen™.
• PE Mouse IgG1, κ Isotype Control, 559320, BD Pharmingen™.
• BV421 Mouse Anti-Human IL-17A, 562933, BD Horizon™.
• BV421 Mouse IgG1, κ Isotype Control, 562438, BD Horizon™.
• FITC Mouse Anti-Human IL-6, 340526, BD FastImmune™.
• FITC Mouse IgG1, κ Isotype Control, 349041, BD™.
• PE/Cyanine7 Anti-Human LAP (TGF-β1), 349610, BioLegend^®^.
• PE/Cyanine7 Mouse IgG1, κ Isotype Control, 400125, BioLegend^®^.

### Statistical methods

Means obtained from each group were compared using a one-way ANOVA. Followed by Kruskal–Wallis or Friedman test for *post hoc* analysis to explore which group means are significantly different from others. The following designations were used in the figures: *: *p* < 0.05, **: *p* < 0.01, ***: *p* < 0.001, ****: *p* < 0.0001 and non-significant: ns. The statistical tests and the graphs were performed using Graph Pad Prism software.

## Results

### Generation of a modified truncated sema3A

**FIGURE 1:**
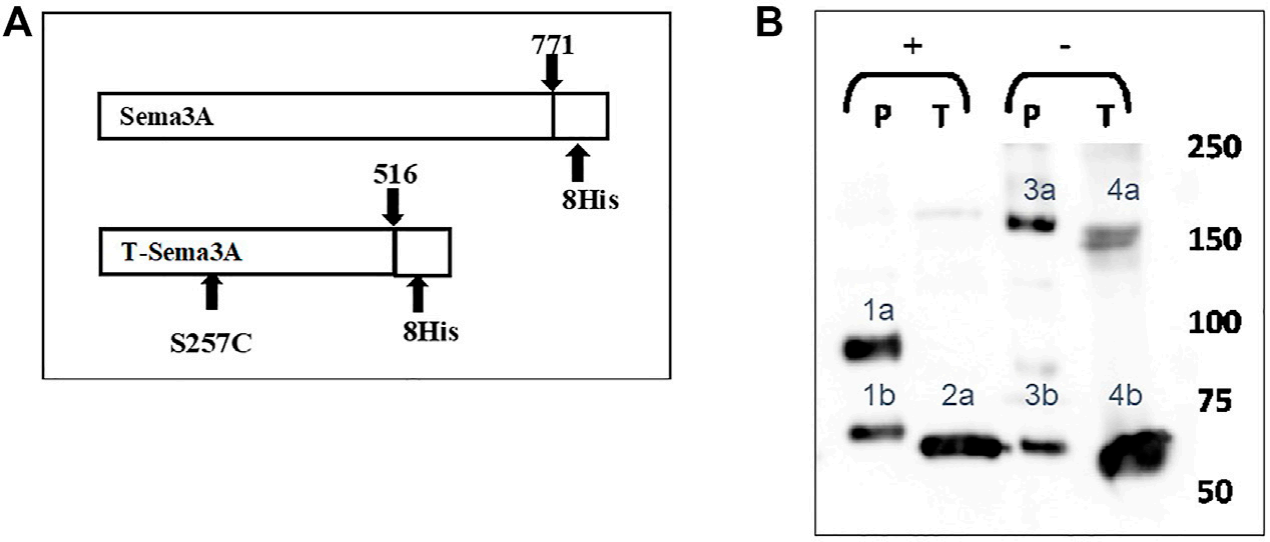
Generation of a modified truncated sema3A **(A)**. The sequence of truncated sema3A (lower scheme) is a modified construct of the original/parental sema3A (upper scheme). The new construct truncated at amino acid 516 of the original sema3A with a point mutation at position 257, which exchanged a serine into a cysteine residue (S257C mutation), with 8xHis epitope tags at the C terminal. **(B)**. The culturing medium of HEK293 cells expressing either P-sema3A or T-sema3A was collected and purified on a nickel affinity column to produce purified parental (P)-sema3A and truncated (T)-sema3A. The purified proteins were loaded on SDS-PAGE in the presence of Dithiothreitol reducing agent (+) or without (-). Then the membrane was immunoblotted with anti-human sema3A polyclonal antibody directed against the N-terminal (26-771 amino acids), followed by anti-goat HRP antibody. The bound antibodies were visualized using the EZ-ECL method and the blots were viewed by ImageQuant LAS 4000 machine and analyzed using ImageQuant TL Analysis software. The bands in the blot represent the following: 1a is P-sema3A monomer (∼95 kDa), 2a and 4b are T-sema3A monomers (∼68 kDa). While 4a double band is T-sema3A homodimer (∼140 kDa) and 3a is P-sema3A dimer (∼160 kDa). Bands 1b and 3b are cleavage fragments of P-sema3A by furin protease.

Our aim was to generate a modified sema3A able to activate CD72 but lacking the ability to bind the NRP-1 receptor. Therefore, based on the crystallography structure of the sema3A-NRP-1 binding module, we produced a truncated sema3A that lacked the C-terminal NRP-1 binding domain downstream of the amino acid 516 (base 1751) (Antipenko et al., 2003; Lee et al., 2003; Janssen et al., 2012). Whereas the active form of sema3A is a homodimer, and because the truncated C-terminal domain contains cysteines required for sema3A dimerization, we exchanged a serine residue at position 257 (base 770) with a cysteine residue to create an artificial dimerization site. Finally, we added 8xHis epitope tag in frame upstream to a stop codon to facilitate the purification of this molecule, which we named Truncated-sema3A (T-sema3A) (Figure 1A). Later, the cDNA encoding the T-sema3A, was transfected using a viral vector, to generate 293HEK cells stably expressing T-sema3A.

To purify Parental (P)-sema3A or T-sema3A proteins, the culturing medium of HEK293 cells expressing either P-sema3A or T-sema3A was collected and loaded on a nickel affinity column. The purified proteins were subjected to SDS-PAGE and immunoblotted with polyclonal antibody directed against the N-terminal of P-sema3A (26-771 amino acids). In the presence of a reducing agent, we verified (in Figure 1B) that the size of the T-sema3A monomer is ∼68 kDa (bands 2a and 4b), while that of P-sema3A is ∼95 kDa (band 1a). Moreover, in order to confirm the homodimer formation of T-sema3A, we loaded the purified protein on SDS-PAGE without a reducing agent and verified that the T-sema3A is indeed able to form a homodimer, presented as ∼140 kDa double band (band 4a). Of importance to mention that the cleavage of P-sema3A by furin protease produces ∼70 kDa fragments (bands 1b and 3b), and P-sema3A dimer is∼160 kDa (band 3a) (Figure 1B).

### Truncated sema3A is unable to induce NRP-1 mediated signaling in endothelial cells

**FIGURE 2:**
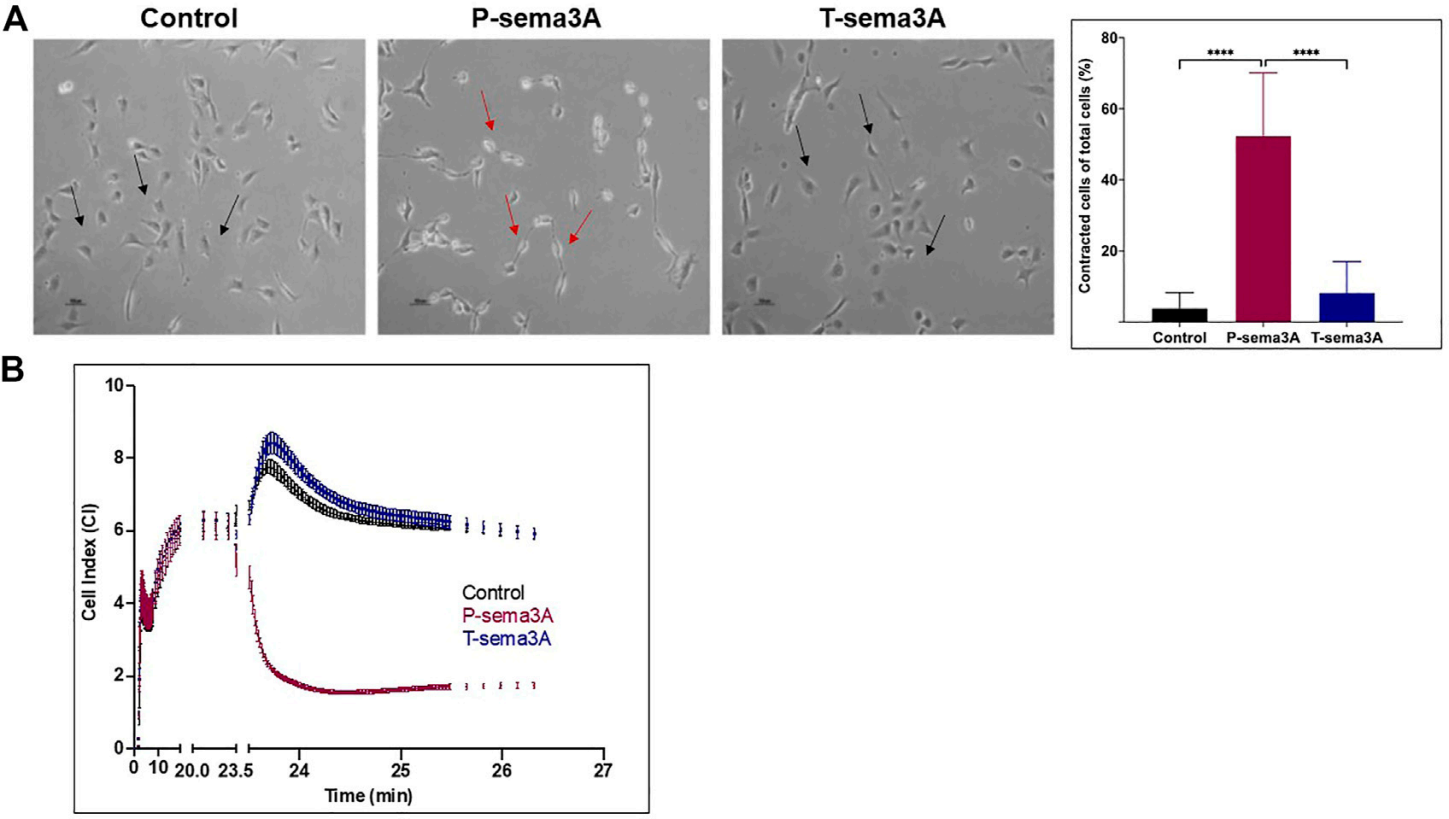
Truncated sema3A is unable to induce NRP-1 mediated signaling in endothelial cells **(A)**. Cell contraction assay was performed on HUVEC cells in the presence of 200 ng/mL of purified P-sema3A or T-sema3A or an appropriate amount of a control elution buffer for 30 min at 37°C. After the incubation time, the cells were photographed using a phase-contrast inverted microscope: A round shape is the natural morphology of the HUVEC (black arrow), while as a result of sema3A, the cytoskeleton collapsed, and the cells lost their round shape (red arrows). The quantification graphs represent the percentage of contracted cells from total cells (N = 8 independent experiments). The results were analyzed using Kaluza software, and the statistical significance was calculated using one-way ANOVA and Kruskal–Wallis test (**** = p-value < 0.0001). **(B)**. Quantitative real-time contraction assay was performed on HUVEC cells placed in an xCELLigence real-time cell analyzer. At time 0, 200 ng/mL of purified P-sema3A or T-sema3A or an appropriate amount of a control elution buffer was added to the wells and the electrical impedance through the E-plates electrodes (which displayed as Cell Index (CI)) was measured for 30 min.

To verify that T-sema3A is unable to activate NRP-1 mediated signal transduction, we performed a cell contraction assay. Human umbilical vein-derived endothelial cells (HUVEC) respond to sema3A by contraction mediated by the NRP-1 receptor (Guttmann-Raviv et al., 2007). P-sema3A was indeed able to induce the collapse of the cytoskeleton of endothelial cells, resulting in cell contraction compared to the control (52.34 ± 17.77 vs 3.84 ± 4.36, *p* < 0.0001, mean ± SD (%)). However, T-sema3A failed to induce cell contraction (8.21 ± 8.77, *p* < 0.0001, mean ± SD (%)) (Figure 2A). Moreover, using real-time cell analyzer RTCA we also demonstrated that the addition of P-sema3A induced a reduction of electrical impedance through the E-plates, indicating that P-sema3A contract HUVEC, while T-sema3A acts as the control and did not cause such a change (Figure 2B). Both assays demonstrate that T-sema3A is unable to activate NRP-1 mediated signal transduction.

### Truncated sema3A specifically binds CD72

**FIGURE 3:**
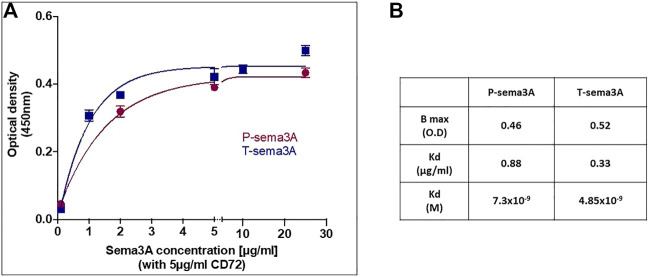
Truncated sema3A can interact with CD72 **(A)**. The ELISA plate was coated with 5 μg/mL purified CD72 and increasing concentrations of purified P-sema3A or T-sema3A were added (0-25 μg/mL). Followed by polyclonal goat anti-human semaphorin3A antibody (directed against the N-terminal of sema3A (26-771 amino acids), then by anti-goat horseradish peroxidase antibody, TMB-Plus substrate chromogen, and a stop solution (10% H2SO4). The optical density (O.D.) was measured at 450nm and 360 nm using an ELISA reader and Gen5 software, and the O.D. of each sample was calculated as O.D. sample minus O.D. background (O.D. background = 0.073). The binding curves representing the optical density as a function of P- or T-sema3A concentration were drawn using GraphPad Prism software. **(B)**. The Kd (equilibrium dissociation constant) and Bmax (maximal binding) were analyzed according to one site total binding equation using GraphPad Prism software.

To show that T-sema3A indeed binds CD72, we performed a binding assay between P-sema3A, T-sema3A, and CD72 recombinant proteins using homemade ELISA, in which increasing concentrations of P- or T-sema3A were added to a CD72 pre-coated plate. The binding curves indicate direct interactions between CD72 and both parental and truncated sema3A (Figure 3A). Moreover, the one site total binding equations show that T-sema3A binds CD72 with a higher affinity than the P-sema3A (Kd ∼4.85 × 10^−9^ M vs. ∼7.3 × 10^−9^ M, respectively) (Figure 3B). Taken together, this experiment suggests that T-sema3A indubitably binds CD72 receptor.

### Truncated sema3A is a stable protein

**FIGURE 4:**
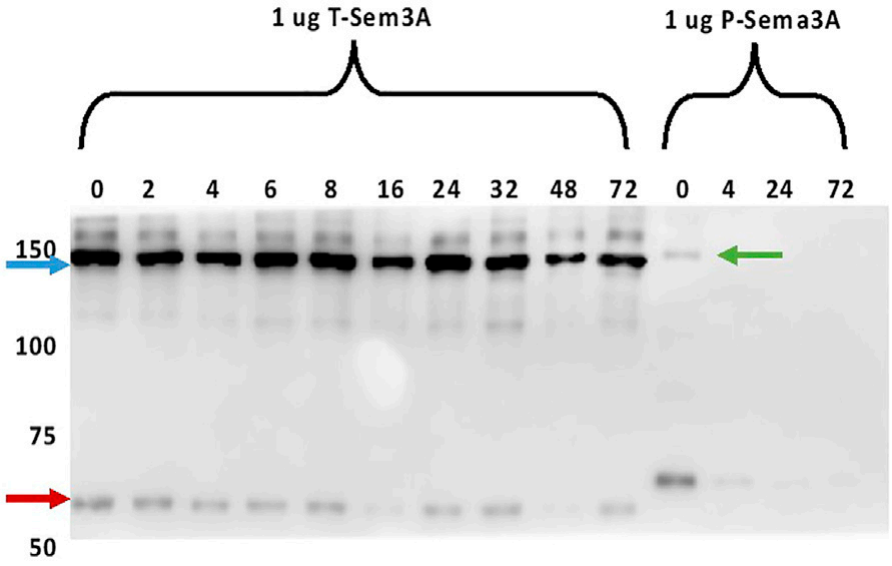
Truncated sema3A is a stable protein. One µg of purified P- and T-sema3A were incubated in 100 µL elution buffer in a 96-well plate at 37°C for 0-72 h. At the indicated time point, proteins were collected, and a sample buffer (without Dithiothreitol redox reagents) was added. All the samples were loaded on SDS-PAGE gel, then the membrane was immunoblotted with anti-human sema3A polyclonal antibody directed against the N-terminal (26-771 amino acids), followed by anti-goat HRP antibody. The bound antibodies were visualized using the EZ-ECL method and the blots were viewed by ImageQuant LAS 4000 machine and analyzed using ImageQuant TL Analysis software. The blue arrow points to the T-sema3A dimer (∼140 kDa), while the red arrow points to T-sema3A monomer (∼68 kDa), and the green arrow points to the P-sema3A dimer (∼160 kDa).

To evaluate the stability of T-sema3A as a dimer at 37°C, we incubated 1 µg of purified P- and T-sema3A at a 37°C incubator for 0-72 h. At the indicated time point, proteins were collected, and a sample buffer (without Dithiothreitol redox reagents) was added. As can be shown (in Figure 4), in the western blot analysis, no differences were observed in the band intensity and band size of T-sema3A dimer (∼140kDa, blue arrow) at all indicated time points, which is not the same phenomena in regard with the P-sema3A (green arrow), indicating that the dimer form of T-sema3A but not P-sema3A remains complete, with no dissociation fractions (Figure 4).

### Truncated sema3A increases regulatory cytokines and decreases pro-inflammatory cytokines in CD3+T lymphocytes

**FIGURE 5:**
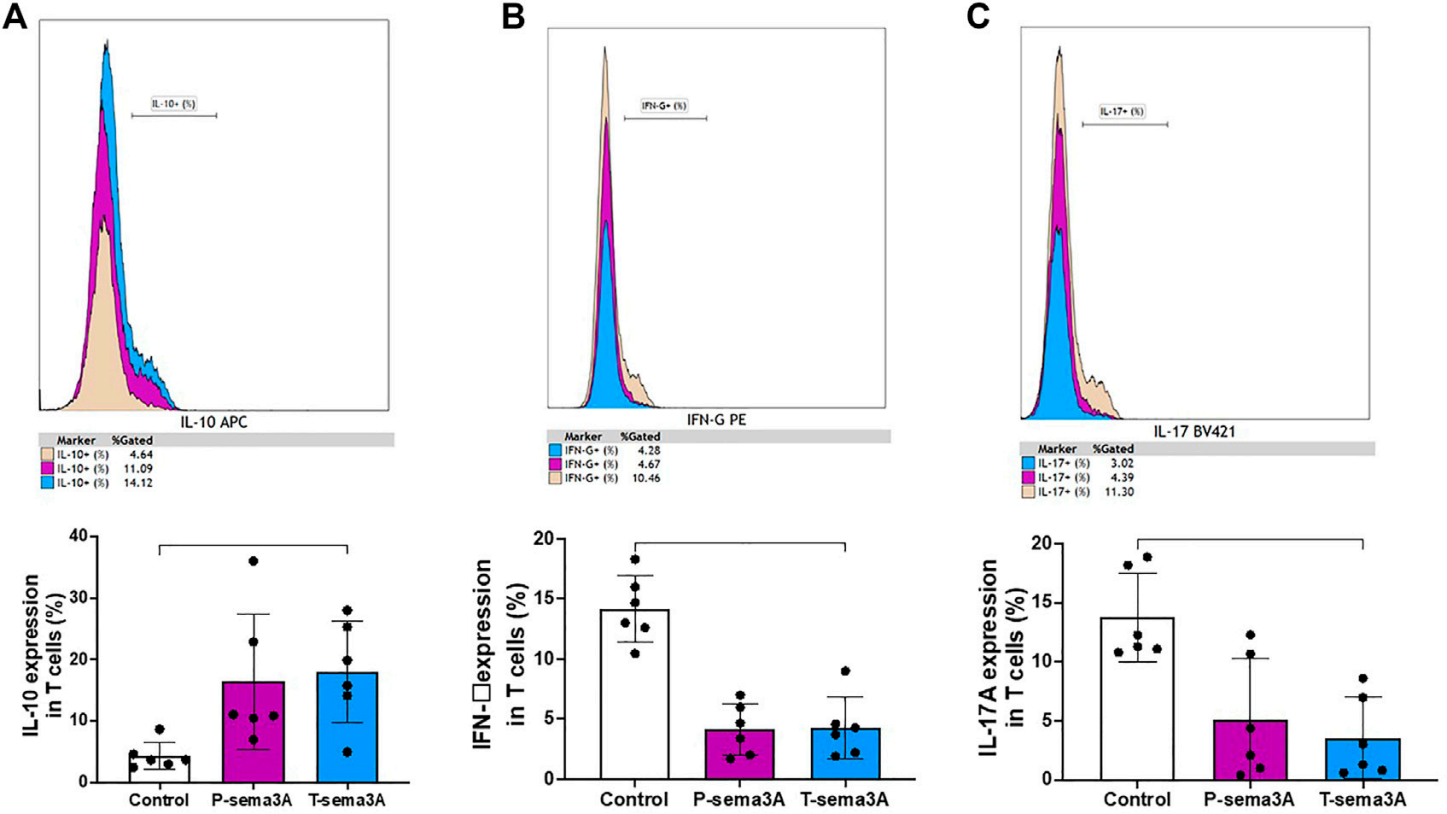
Truncated sema3A induces regulatory pattern in CD3+T lymphocytes. Primary CD3+T cells were isolated from PBMCs using MACS microbeads and MS column. The cells were cultured in 12-well plates, pre-coated with 10 μg/mL anti-CD3 for 4 h at 37 °C, in addition to 1 μg/mL anti-CD28, for 24 h at 37 °C. After 24 h, 1 μg/mL of P- or T-sema3A or an appropriate amount of a control elution buffer was added for an extra 24 h at 37 °C. On the day of the experiment, cells were stained with fluorescence antibodies and the expression of the following cytokines was evaluated in a NAVIOUS EX flow cytometer. The results were analyzed using Kaluza software, and the statistical significance was calculated using one-way ANOVA and Friedman test (*= *p*-value<0.05). **(A)**. IL-10 **(B)**. IFN-γ **(C)**. IL-17A.

Stimulation of primary purified CD3+T cells with either P-sema3A or T-sema3A induced a high level of expression of IL-10 compared to the control (16.04 ± 11.03 and 18.02 ± 8.32 vs 4.37 ± 2.24, p = 0.0117, mean ± SD (%), respectively) (Figure 5A). In contrast, low levels of IFN-γ were observed following P-sema3A or T-sema3A stimulation compared to the control (4.13 ± 2.15 and 4.28 ± 2.56 vs 14.18 ± 2.77, p = 0.0281, mean ± SD (%), respectively) (Figure 5B). The same pattern of lowered pro-inflammatory cytokine IL-17A secretion was noticed following P-sema3A or T-sema3A stimulation (5.15 ± 5.13 and 3.55 ± 3.44 vs 13.77 ± 3.75, p = 0.0117, mean ± SD (%), respectively) (Figure 5C). These results show that the biological effect of truncated sema3A on CD3+T cells is equal to the effect of parental sema3A, since both increase the expression of the regulatory cytokine IL-10 and decrease the expression of pro-inflammatory cytokines.

### Truncated sema3A increases the expression of regulatory cytokines in B-lymphocytes

**FIGURE 6:**
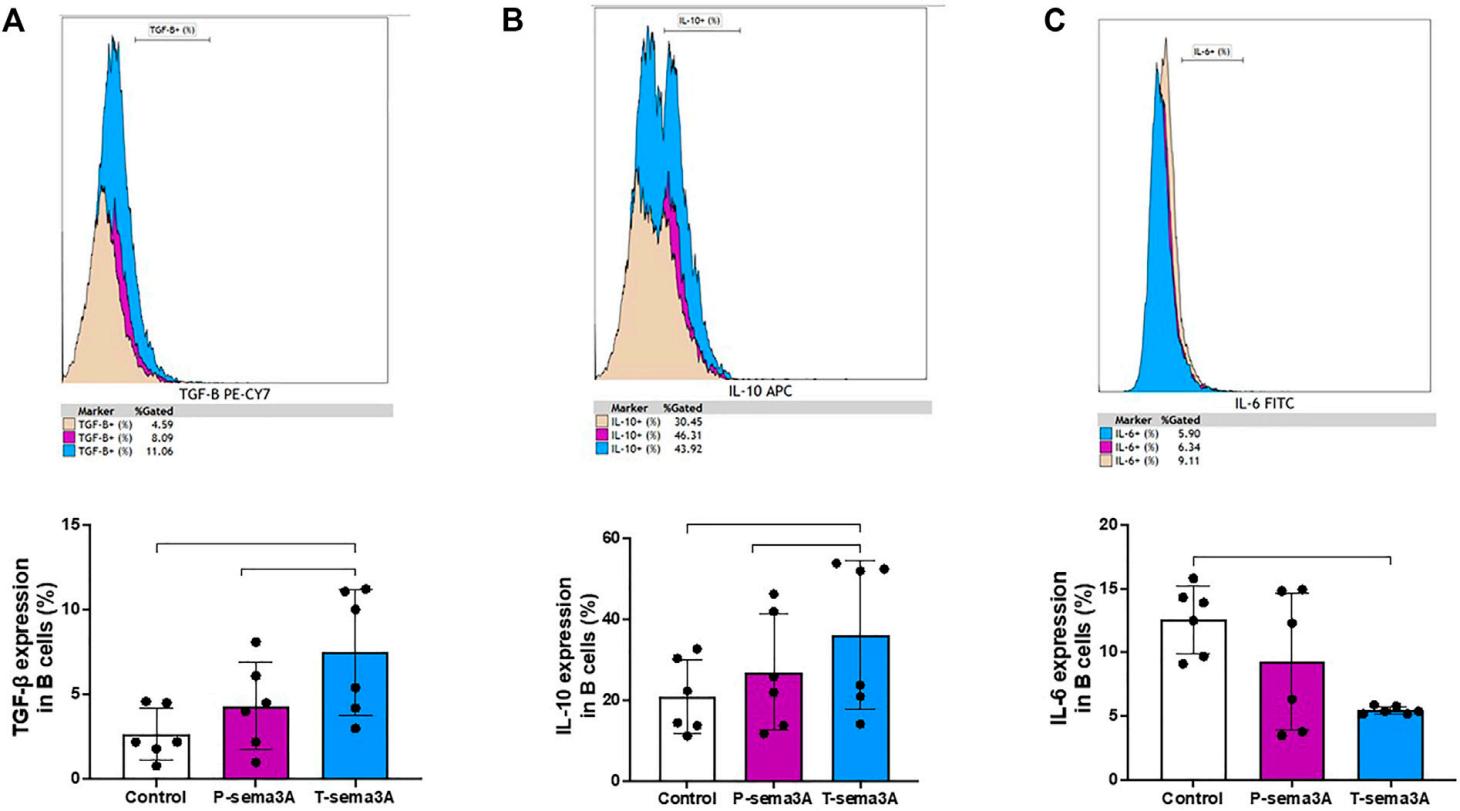
Truncated sema3A induces regulatory pattern in B lymphocytes. CD22^+^ B cells were isolated from PBMCs using MACS microbeads and MS column. The positively isolated B cells were cultured in 12-well plates and activated with 1 µM TLR9 agonist-CpG-ODN and 5 μg/mL CD40L at 37 °C for 24 h. After 24 h, 1 μg/mL of P- or T-sema3A or the appropriate amount of a control elution buffer was added for an additional 24 h at 37 °C. On the day of the experiment, cells were stained with fluorescence antibodies and the expression of the following cytokines was evaluated in a NAVIOUS EX flow cytometer. The results were analyzed using Kaluza software, and the statistical significance was calculated using one-way ANOVA and Friedman test (*= *p*-value<0.05 and ** = *p*-value<0.01). **(A)**. TGF-β1 **(B)**. IL-10 **(C)**. IL-6.

Stimulation of primary purified human B cells with either P-sema3A or T-sema3A induced a high level of expression of TGF-β compared to the control (4.32 ± 2.57 and 7.48 ± 3.69 vs 2.68 ± 1.53, p = 0.0045, mean ± SD (%), respectively) (Figure 6A). Additionally, the same pattern was shown with the anti-inflammatory cytokine IL-10 (26.99 ± 14.33 and 36.24 ± 18.43 vs 20.86 ± 9.17, p = 0.0045, mean ± SD (%), respectively) (Figure 6B). Moreover, statistically this increased level of expression of IL-10 and TGF-β was significantly higher in the T-sema3A than in the P-sema3A treated cells (p = 0.0312 and p = 0.0312, respectively). In contrast, the low secretion of IL-6 was shown following either P-sema3A or T-sema3A stimulation compared to the control (9.27 ± 5.35 and 5.48 ± 0.23 vs 12.55 ± 2.66, p = 0.0281, mean ± SD (%), respectively) (Figure 6C). Moreover, these results show that truncated sema3A is more effective than parental sema3A in increasing the expression of the regulatory cytokines IL-10 and TGF-β and is as effective as parental sema3A in decreasing the expression of the pro-inflammatory cytokine IL-6.

## Discussion

The significance of sema3A in being a regulatory master of upstream signaling pathways in immune cells and playing a role in regulating immune-mediated inflammation is widely reported. When sema3A was added to co-culture of dendritic and T cells, it significantly inhibited allogeneic T-cell proliferation (Lepelletier et al., 2006). The addition of sema3A to activated B cells of SLE patients decreased the expression of TLR-9, suggesting its role in the process of suppressing B-cell over-activity (Vadasz et al., 2014). One of the important regulatory receptors on B cells is CD72, the ligation of which was found to down-regulate B-cell receptor-related signaling, thereby maintaining self-tolerance. The up regulation of CD72 on B cells (of both normal individuals and patients suffering from SLE) following their co-culture with sema3A was reported in our seminal study (Vadasz et al., 2014), suggesting that sema3A is a ligand for CD72 on B cells. Subsequently, we could definitely show that sema3A binds CD72, resulting in the restoration of B cell homeostasis and self-tolerance (Eiza et al., 2022). With this in mind, the majority of studies do support the idea of Sema3A being a regulatory molecule. However, some papers reported on the increased expression of sema3A in some tissues such as in the tubule of SLE patients and in the synovium of RA patients, which was suggested to be an exacerbating factor in these diseases (Liu et al., 2018). However, we do believe that this is a compensatory mechanism aiming to prevent autoimmunity rather than an exacerbating factor. With this in mind, the majority of studies do support the idea of sema3A being a regulatory molecule.

In our current study, we demonstrate our new results of creating a new synthetic sema3A (T-sema3A), which is easy to produce, but most importantly, is a stable molecule and of a high binding affinity to CD72. The exclusion of binding T-sema3A to NRP-1 expressing cells is highly appreciated since such binding might potentially induce unwanted neurotoxicity in the peripheral and systemic neural systems. For this reason, the development of this synthetic T-sema3A has great therapeutic potential and is worth assessing. In this respect, we first show that T-sema3A has a better binding ability to CD72, which certainly may induce a better regulatory function. Aiming to obtain a beneficial therapeutic agent, we recognize that our results possess a stable compound that could be induced *in-vivo* and maintain a long-standing effect. Therefore, our functional results of T-sema3A effect in both T and B-lymphocytes are of significant importance. Once T-sema3A’ ability to increase the expression of regulatory cytokines along with their ability to decrease pro-inflammatory cytokines will be also proven in *in-vivo* immune-mediated and autoimmune diseases mice models. Future studies should then be performed to strengthen the stability of T-sema3A *in vivo* (first animal models and then human models).

## Data Availability

The raw data supporting the conclusion of this article will be made available by the authors, without undue reservation.

## References

[B1] Antipenko A. Himanen J. P. van Leyen K. Nardi-Dei V. Lesniak J. Barton W. A. (2003). Structure of the Semaphorin-3A receptor binding module. Neuron 39, 589–598. 10.1016/S0896-6273(03)00502-6 12925274

[B2] Catalano A. (2010). The neuroimmune semaphorin-3A reduces inflammation and progression of experimental autoimmune arthritis. J. Immunol. 185, 6373–6383. 10.4049/jimmunol.0903527 20937848

[B3] Eiza N. Sabag A. D. Kessler O. Neufeld G. Vadasz Z. (2022). CD72-semaphorin3A axis: A new regulatory pathway in systemic lupus erythematosus. J. Autoimmun. 134, 102960. 10.1016/J.JAUT.2022.102960 36470209

[B4] Guttmann-Raviv N. Shraga-Heled N. Varshavsky A. Guimaraes-Sternberg C. Kessler O. Neufeld G. (2007). Semaphorin-3A and semaphorin-3F work together to repel endothelial cells and to inhibit their survival by induction of apoptosis. J. Biol. Chem. 282, 26294–26305. 10.1074/JBC.M609711200 17569671

[B5] Jaffe E. A. Nachman R. L. Becker C. G. Minick C. R. (1973). Culture of human endothelial cells derived from umbilical veins. Identification by morphologic and immunologic criteria. J. Clin. Invest. 52, 2745–2756. 10.1172/JCI107470 4355998 PMC302542

[B6] Janssen B. J. C. Malinauskas T. Weir G. A. Cader M. Z. Siebold C. Jones E. Y. (2012). Neuropilins lock secreted semaphorins onto plexins in a ternary signaling complex. Nat. Struct. Mol. Biol. 19, 1293–1299. 10.1038/NSMB.2416 23104057 PMC3590443

[B7] Lee C. C. Kreusch A. McMullan D. Ng K. Spraggon G. (2003). Crystal structure of the human neuropilin-1 b1 domain. Structure 11, 99–108. 10.1016/S0969-2126(02)00941-3 12517344

[B8] Li D. H.-H. Winslow M. M. Cao T. M. Chen A. H. Davis C. R. Mellins E. D. (2008). Modulation of peripheral B cell tolerance by CD72 in a murine model. Arthritis Rheum. 58, 3192–3204. 10.1002/art.23812 18821699 PMC2790383

[B9] Liu L. N. Li X. M. Ye D. Q. Pan H. F. (2018). Emerging role of semaphorin-3A in autoimmune diseases. Inflammopharmacology 26, 655–665. 10.1007/S10787-018-0484-Y 29696565

[B10] Moreau-Fauvarque C. Kumanogoh A. Camand E. Jaillard C. Barbin G. Boquet I. (2003). The transmembrane semaphorin Sema4D/CD100, an inhibitor of axonal growth, is expressed on oligodendrocytes and upregulated after CNS lesion. J. Neurosci. 23, 9229–9239. 10.1523/jneurosci.23-27-09229.2003 14534257 PMC6740837

[B11] Neufeld G. Gospodarowicz D. (1988). Identification of the fibroblast growth factor receptor in human vascular endothelial cells. J. Cell Physiol. 136, 537–542. 10.1002/JCP.1041360321 2844833

[B12] Neufeld G. Kessler O. (2008). The semaphorins: Versatile regulators of tumour progression and tumour angiogenesis. Nat. Rev. Cancer 8, 632–645. 10.1038/nrc2404 18580951

[B13] Nitschke L. Tsubata T. (2004). Molecular interactions regulate BCR signal inhibition by CD22 and CD72. Trends Immunol. 25, 543–550. 10.1016/j.it.2004.08.002 15364057

[B14] Shapiro M. Akiri G. Chin C. Wisnivesky J. P. Beasley M. B. Weiser T. S. (2013). Wnt pathway activation predicts increased risk of tumor recurrence in patients with stage I nonsmall cell lung cancer. Ann. Surg. 257, 548–554. 10.1097/SLA.0B013E31826D81FD 23011390 PMC3546156

[B15] Shirvan A. Ziv I. Fleminger G. Shina R. He Z. Brudo I. (1999). Semaphorins as mediators of neuronal apoptosis. J. Neurochem. 73, 961–971. 10.1046/j.1471-4159.1999.0730961.x 10461885

[B16] Takegahara N. Kumanogoh A. Kikutani H. (2005). Semaphorins: A new class of immunoregulatory molecules. Philosophical Trans. R. Soc. B Biol. Sci. 360, 1673–1680. 10.1098/rstb.2005.1696 PMC1569539 16147531

[B17] Tamagnone L. Comoglio P. M. (2000). Signalling by semaphorin receptors: Cell guidance and beyond. Trends Cell Biol. 10, 377–383. 10.1016/S0962-8924(00)01816-X 10932095

[B18] Tessler S. Neufeld G. (1990). Basic fibroblast growth factor accumulates in the nuclei of various bFGF-producing cell types. J. Cell Physiol. 145, 310–317. 10.1002/JCP.1041450216 2246329

[B19] Vadasz Z. Attias D. Kessel A. Toubi E. (2010). Neuropilins and semaphorins - from angiogenesis to autoimmunity. Autoimmun. Rev. 9, 825–829. 10.1016/j.autrev.2010.07.014 20678594

[B20] Vadasz Z. Ben-Izhak O. Bejar J. Sabo E. Kessel A. Storch S. (2011). The involvement of immune semaphorins and neuropilin-1 in lupus nephritis. Lupus 20, 1466–1473. 10.1177/0961203311417034 21951945

[B21] Vadasz Z. Haj T. Balbir A. Peri R. Rosner I. Slobodin G. (2014). A regulatory role for CD72 expression on B cells in systemic lupus erythematosus. Semin. Arthritis Rheum. 43, 767–771. 10.1016/j.semarthrit.2013.11.010 24461079

[B22] Vadasz Z. Haj T. Halasz K. Rosner I. Slobodin G. Attias D. (2012). Semaphorin 3A is a marker for disease activity and a potential immunoregulator in systemic lupus erythematosus. Arthritis Res. Ther. 14, R146. 10.1186/ar3881 22697500 PMC3446531

[B23] Varshavsky A. Kessler O. Abramovitch S. Kigel B. Zaffryar S. Akiri G. (2008). Semaphorin-3B is an angiogenesis inhibitor that is inactivated by furin-like pro-protein convertases. Cancer Res. 68, 6922–6931. 10.1158/0008-5472.CAN-07-5408 18757406

[B24] Zhang Y. Liu B. Ma Y. Jin B. (2013). Sema 4D/CD100-plexin B is a multifunctional counter-receptor. Cell Mol. Immunol. 10, 97–98. 10.1038/cmi.2012.65 23262975 PMC4003050

